# Magnetic Resonance Spectroscopy in the Evaluation of Biopsy-Indeterminate Primary Central Nervous System Lymphoma: A Case Report

**DOI:** 10.7759/cureus.91539

**Published:** 2025-09-03

**Authors:** Mark D Marino, William Kardasis, John P Mader, Michael Syrett, Shamseldeen Y Mahmoud

**Affiliations:** 1 Radiology, Saint Louis University School of Medicine, Saint Louis, USA

**Keywords:** corticosteroids, diffuse large b cell lymphoma, immunohistochemistry, magnetic resonance imaging (mri), magnetic resonance spectroscopy (mrs), neuroradiology, primary central nervous system lymphoma (pcnsl), stereotactic brain biopsy

## Abstract

Primary central nervous system lymphoma (PCNSL) is a rare, aggressive extra-nodal non-Hodgkin lymphoma restricted to the central nervous system without systemic involvement at diagnosis. PCNSL can present with a wide array of symptoms, including cognitive dysfunction, focal neurologic deficits, or seizures. This case report utilizes a chart review of the history, physical examination, laboratory tests, imaging findings, and pathology reports recorded during this patient’s hospital encounters. We present the case of a middle-aged woman with a past medical history of traumatic brain injury and seizures with incidental neuroimaging findings on head computed tomography showing right parietal lobe and subcortical edema. She reported subacute onset of diffuse paresthesia, generalized weakness, severe migraine headaches, poor balance, and falls. She was admitted to the hospital for further evaluation of her neurological symptoms and was started on prophylactic dexamethasone. Magnetic resonance imaging (MRI) demonstrated avidly enhancing lesions in the bilateral cingulate gyri and periventricular regions, right frontoparietal region, right temporal lobe, corpus callosum, hypothalamus, and basal ganglia, with vasogenic edema and midline shift. Magnetic resonance spectroscopy (MRS) yielded an elevated choline-to-creatinine ratio, decreased N-acetylaspartate peak, normal myoinositol, and a prominent lipid peak - findings consistent with PCNSL. Steroids were continued after discharge to treat cerebral edema, and a stereotactic biopsy performed 28 days later was non-diagnostic. Subsequent brain MRI showed marked disease regression, and cerebrospinal fluid analysis failed to confirm a diagnosis. Steroids were discontinued, and a repeat biopsy was performed two months later after symptomatic and radiologic progression. Pathology analysis determined a diagnosis of diffuse large B-cell lymphoma. This case highlights the classic imaging and MRS findings associated with PCNSL and underscores the diagnostic challenges posed by continued corticosteroid use, which can lead to false-negative biopsy, cytology, and flow cytometry. Avoiding unnecessary delays in diagnosing PCNSL is critical, as timely initiation of targeted chemotherapy improves outcomes. When MRI and MRS findings support a diagnosis of PCNSL, physicians may opt for early biopsy and avoid steroids even if cerebral edema is present. This case also supports the growing role of MRS as a valuable adjunct in characterizing CNS lesions - especially when biopsy is non-diagnostic or contraindicated. Further research and standardization of MRS techniques may enhance its utility in non-invasively diagnosing PCNSL in the future.

## Introduction

Primary central nervous system lymphoma (PCNSL) is a rare, aggressive, extra-nodal non-Hodgkin lymphoma confined to the brain, spinal cord, leptomeninges, or eyes without systemic involvement at diagnosis. It arises from the malignant transformation of late germinal center B-cells and is marked by aberrant B-cell receptor and Toll-like receptor signaling, constitutive NF-κB activation, somatic hypermutation, and immune escape via major histocompatibility complex downregulation and a suppressive tumor microenvironment [1-3]. Patients present most commonly with cognitive dysfunction, psychomotor slowing, personality changes, and disorientation; less common late-stage symptoms include raised intracranial pressure, focal neurological deficits, cranial nerve dysfunction, ataxia, and seizures [1,4,5]. Cerebrospinal fluid (CSF) analysis is often normal in cases of PCNSL but may reveal elevated protein, mild pleocytosis, and, less commonly, hypoglycorrhachia [6,7]. Flow cytometry may be positive for CD10, CD19, CD20, BCL-2, BCL-6, and PAX-5 [8-10]. The most common type of PCNSL is diffuse large B-cell lymphoma, making up 95% of PCNSL cases [11,12].

Magnetic resonance imaging (MRI) can provide valuable information for the initial detection of PCNSL. MRI findings for PCNSL include well-defined homogeneous contrast-enhancing lesions, T2 hypointense or isointense lesions, and restricted diffusion; vasogenic edema and mass effect are commonly present [13]. Common locations for PCNSL lesions include the frontal lobe, ventricular areas, cingulate gyrus, and basal ganglia [14-16]. Magnetic resonance spectroscopy (MRS) adds diagnostic value by revealing hallmark findings such as elevated choline-to-creatine ratios, decreased N-acetylaspartate, and prominent lipid peaks, even without central necrosis [17-19]. Myoinositol peaks are typically within normal limits in PCNSL [20]. Metabolic characterization with MRS can distinguish PCNSL from high-grade gliomas or tumefactive demyelinating lesions and enhance biopsy targeting to reduce diagnostic delay [17,21]. Timely recognition of these imaging features is essential given the aggressive nature of PCNSL and the urgency of initiating targeted therapy.

The typical treatment for PCNSL is high-dose methotrexate (HD-MTX)-based chemotherapy, which can be combined with monoclonal antibodies such as rituximab or nivolumab, and chemotherapy agents such as cytarabine, procarbazine, and vincristine [22]. Steroids are frequently used to manage cerebral edema and mass effect, but their use prior to diagnostic biopsy is controversial. Corticosteroids can induce rapid tumor lysis and radiographic regression, potentially obscuring histopathologic diagnosis [23-25]. Therefore, steroids should be avoided prior to biopsy unless urgent clinical deterioration necessitates their use. Here, we present a case of a woman with a brain mass that was biopsy-inconclusive due to steroid administration. As a result, neuroimaging played a crucial role in tracking the suspected PCNSL lesions until a final pathology diagnosis was possible. This case highlights the possibilities of MRI and MRS in diagnosing PCNSL when steroids are suspected to cause a false-negative biopsy.

## Case presentation

Recently, a middle-aged female patient with a past medical history of traumatic brain injury and remote seizures presented to a neurosurgery clinic appointment after concerning incidental findings were seen on neuroimaging. A head computed tomography (CT) exam performed at an outside hospital revealed edema of the right parietal lobe and generalized subcortical edema. The patient reported a subacute onset of diffuse paresthesia, generalized weakness, severe migraine headaches, poor balance, and falls. Neurosurgery recommended hospital admission for further evaluation of her neurologic symptoms, with neurosurgery, neurology, and infectious disease consulted to rule out a demyelinating lesion, mass, neoplasm, or infectious process. She was started on prophylactic dexamethasone and received a loading dose of levetiracetam.

The patient was afebrile with stable vital signs on admission. A physical exam revealed a positive Romberg sign. Serum labs were normal, and blood cultures were negative (Table 1). CT of the head demonstrated irregularly shaped right posterior frontal and parietal lobe masses with significant vasogenic edema throughout the right cerebral convexity and approximately 1 or 2 mm of right-to-left midline shift.

**Table 1:** A summary of relevant laboratory findings with reference ranges included Abnormal results are in bold. LDH: lactate dehydrogenase; HIV: human immunodeficiency virus

Lab	Reference range	Patient value
White blood cells	4-10.7 g/dL	6.7
Hemoglobin	11.9-15.8 g/dL	16.7
Platelet count	150-420	267
LDH	125-243 units/L	206
C-reactive protein	<0.5 mg/dL	<0.5
Erythrocyte sedimentation rate	0-20 mm/hour	11
HIV antigen/antibody	Non-reactive	Non-reactive
Hepatitis B core antibody	Non-reactive	Non-reactive
*Treponema pallidum* antibody	Non-reactive	Non-reactive

**Figure 1:**
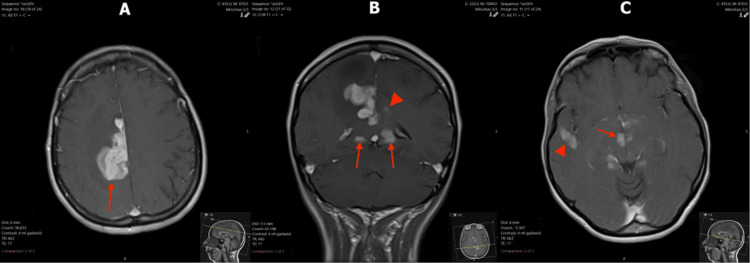
T1 post-contrast MRI images of the brain (A) Axial image with an avidly enhancing right cingulate gyrus/frontoparietal lesion with surrounding vasogenic edema (arrow). (B) Coronal image demonstrates a well-defined, homogeneously enhancing lesion involving the right cingulate gyrus with surrounding vasogenic edema. Additional bilateral lesions adjacent to the occipital horns of both lateral ventricles (arrows) and trace enhancement of the left cingulate gyrus (arrowhead) are also depicted. (C) Axial image with enhancing lesions in the right temporal lobe (arrowhead) and abutting the third ventricle (arrow). MRI: magnetic resonance imaging

**Figure 2:**
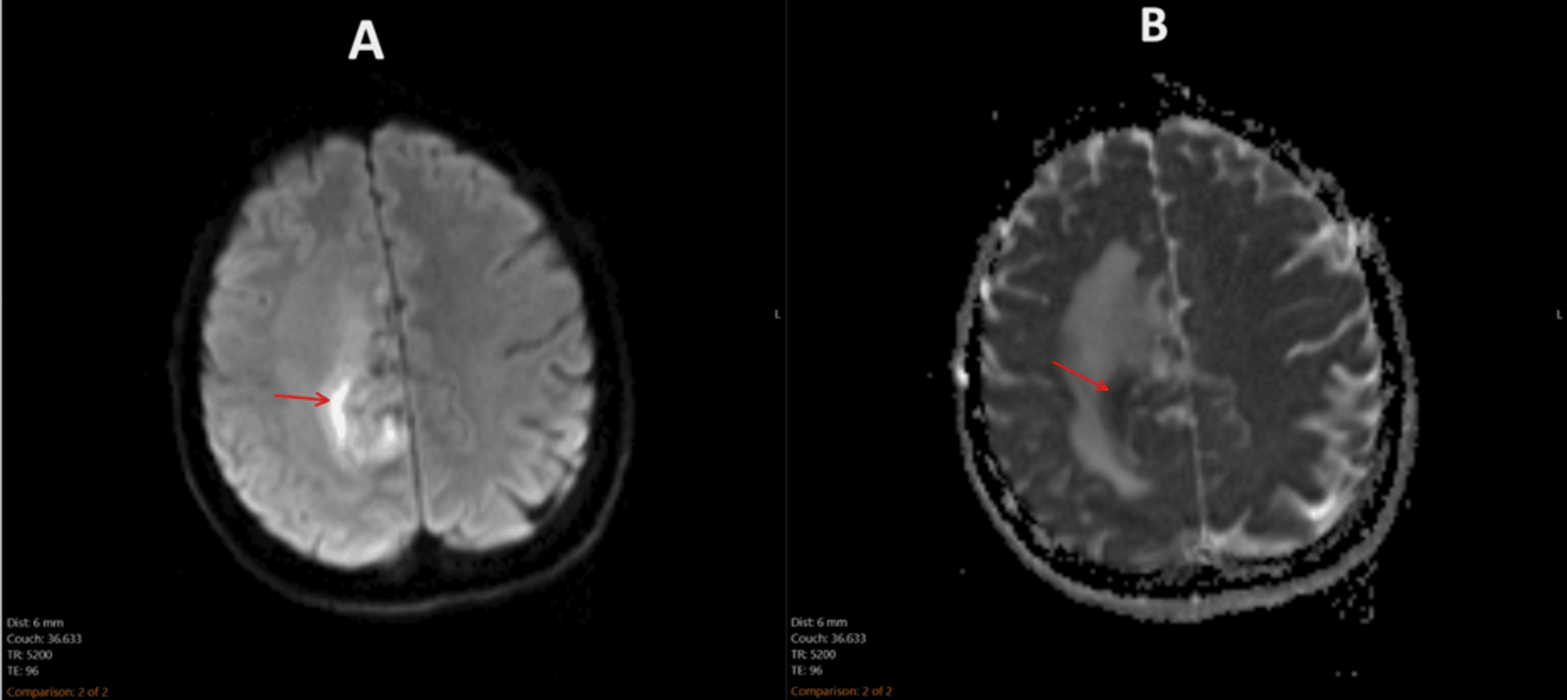
DWI and corresponding ADC mapping of a right frontoparietal lesion (A) DWI demonstrates an intense signal at the right frontoparietal lesion (arrow). (B) Corresponding ADC mapping confirms the presence of true diffusion restriction (arrow). DWI: diffusion-weighted imaging; ADC: apparent diffusion coefficient

**Figure 3:**
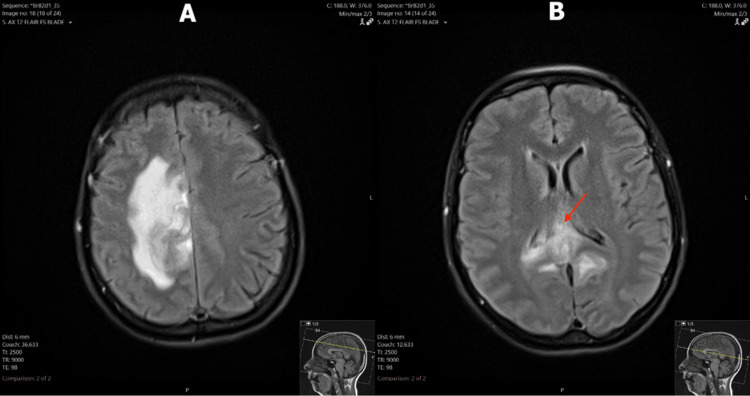
T2-weighted FLAIR axial MRI images of the brain (A) Irregular, heterogeneous FLAIR signal in the right cingulate gyrus/frontoparietal region with adjacent FLAIR hyperintensity representing vasogenic edema. (B) Poorly defined, heterogeneous, mildly hyperintense FLAIR signal in the splenium of the corpus callosum with adjacent subependymal involvement (arrow). FLAIR: fluid-attenuated inversion recovery; MRI: magnetic resonance imaging

**Figure 4:**
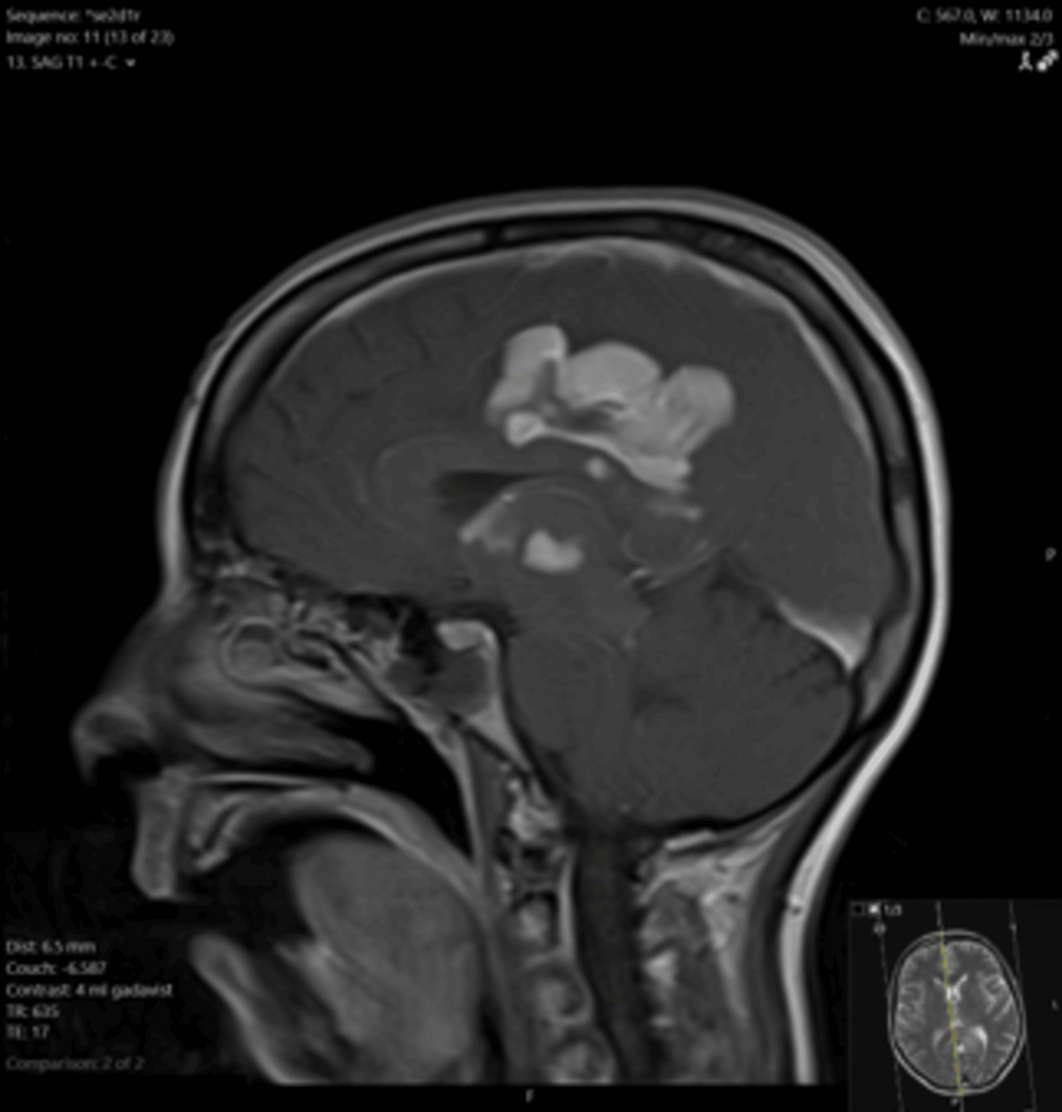
T1 post-contrast sagittal MRI image with avidly enhancing lesions in the right cingulate gyrus, corpus callosum, basal ganglia, and hypothalamus MRI: magnetic resonance imaging

Due to the irregular masses identified on CT, an MRI of the brain was performed. The MRI demonstrated irregular, multifocal, and avidly enhancing lesions in the bilateral cingulate gyri and periventricular regions, right frontoparietal region, and right temporal lobe (Figure 1). An area of restricted diffusion was appreciated in the right frontoparietal region (Figure 2). T2-weighted fluid-attenuated inversion recovery (FLAIR) sequence showed heterogeneous lesions in the right frontoparietal region with adjacent vasogenic edema, and at the splenium of the corpus callosum with subependymal involvement (Figure 3). Large avidly enhancing lesions involving the corpus callosum, basal ganglia, and hypothalamus were also revealed (Figure 4). Mild right-to-left midline shift was seen again. Pertinent negatives included an absence of necrosis or hemorrhage.

**Figure 5:**
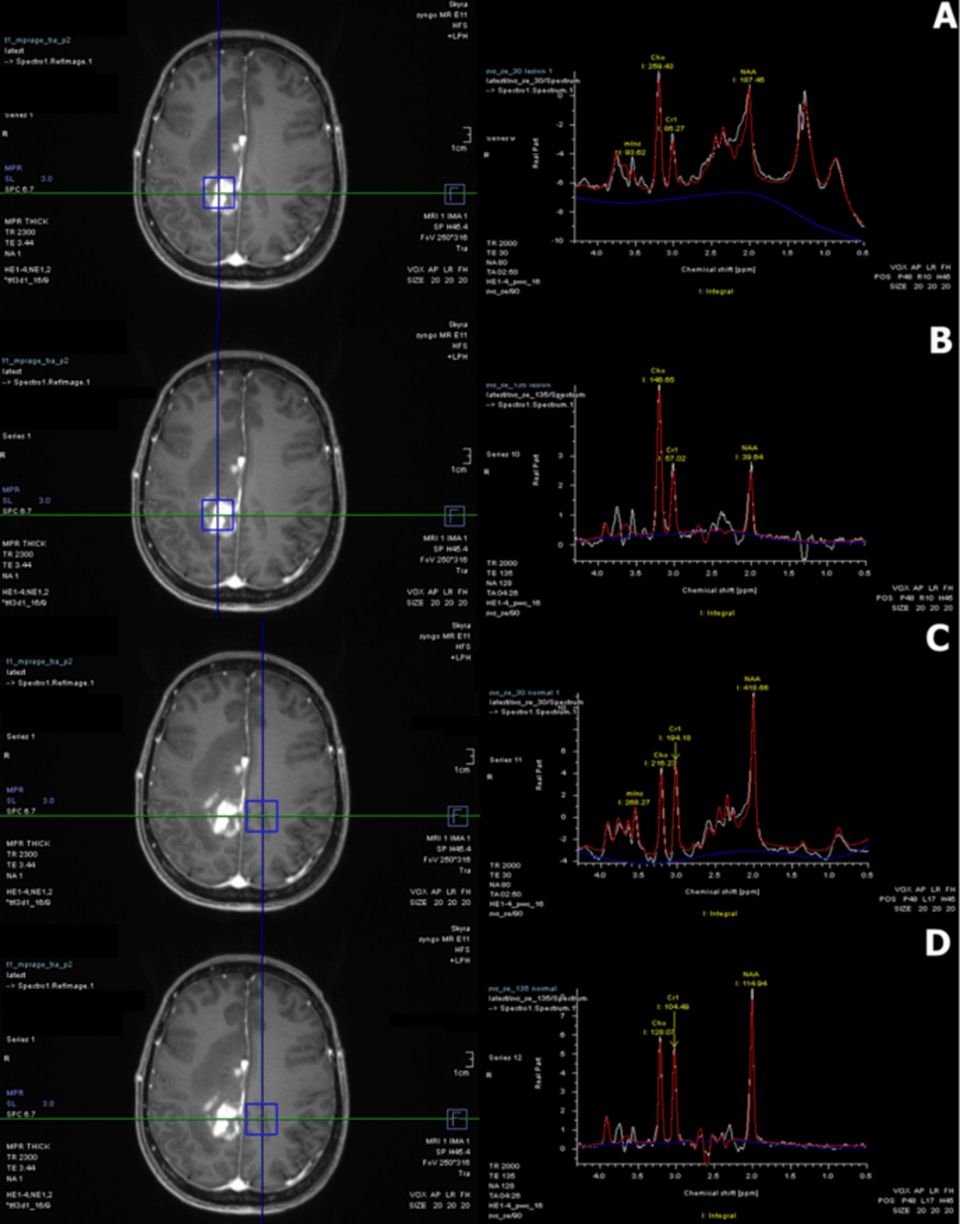
MRS analysis of a right frontoparietal lesion voxel compared to normal brain tissue on the contralateral side (A) MRS analysis of a voxel containing the right frontoparietal lesion with a TE of 30 ms. Mins peak appears normal. The Cho/Cr ratio is elevated. There is a large bimodal lactate-lipid peak from 0.5 to 1.5 ppm. (B) MRS analysis of a voxel containing the right frontoparietal lesion with a TE of 135 ms. The Cho/Cr ratio is elevated. The NAA peak is abnormally small. (C) MRS analysis of a voxel of the normal left frontoparietal region with a TE of 30 ms. Metabolite peaks are within normal limits. (D) MRS analysis of a voxel of the normal left frontoparietal region with a TE of 135 ms. Metabolite peaks are within normal limits. MRS: magnetic resonance spectroscopy; TE: echo time; ms: milliseconds; mins: myoinositol; Cho: choline; Cr: creatinine; ppm: parts per million; NAA: N-acetylaspartate

**Figure 6:**
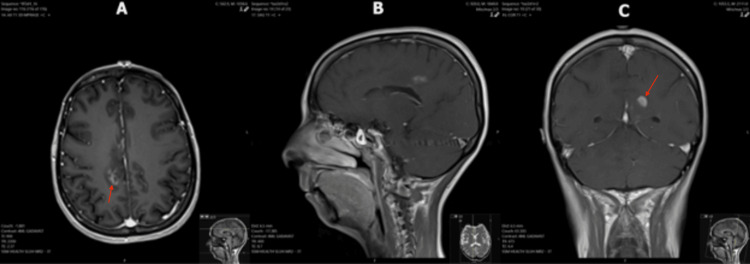
T1 post-contrast MRI images demonstrating significant interval regression of multiple PCNSL lesions after steroid administration (A) Axial image demonstrates an interval decrease in size of the right frontoparietal/cingulate gyrus lesion (arrow) and corresponding reductions in vasogenic edema and mass effect. (B) Sagittal image shows near-complete resolution of the cingulate gyrus, corpus callosum, basal ganglia, and hypothalamus lesions. (C) Coronal image with a small but enlarged lesion in the left cingulate gyrus (arrow). There was complete interval resolution of the previously noted enhancing lesions adjacent to the bilateral occipital horns of the lateral ventricles. MRI: magnetic resonance imaging; PCNSL: primary central nervous system lymphoma

To further characterize these central nervous system (CNS) lesions, MRI spectroscopy was performed with echo times (TE) of 30 and 135 ms. Compared to the contralateral normal, spectroscopy analysis of the right frontoparietal lesion yielded a grossly normal myoinositol peak, an increased choline-to-creatinine ratio, and a decrease in N-acetylaspartate (Figure 5). For the lesion in question, the 30 ms TE myoinositol-to-creatinine ratio measured 1.09, and the 135 ms TE choline-to-creatinine ratio measured 2.57. Meanwhile, the myoinositol-to-creatinine ratio and choline-to-creatinine ratio of the contralateral normal measured 1.38 and 1.23, respectively. There was a large bimodal lactate-lipid peak at 30 ms, but these metabolites appeared normal at 135 ms. These findings suggested a diagnosis of PCNSL, but other CNS neoplasms could not be excluded. Dexamethasone was continued after discharge to reduce cerebral edema and alleviate symptoms before stereotactic biopsy. The patient underwent a right craniotomy for biopsy 28 days after the original MRI. The neurosurgery team targeted the right temporal lobe lesion (Figure 1C) after preoperative CT, but the biopsy was non-diagnostic. A repeat brain MRI and craniotomy for biopsy were scheduled to be performed in one month. When the patient returned to the hospital for repeat imaging, the brain MRI revealed a marked decrease in the size of the enhancing CNS lesions and corresponding vasogenic edema (Figure 6). The previously noted right frontoparietal lesion was reduced in size, and the periventricular lesions were not present on this scan. Enlargement of the enhancing lesion of the left cingulate gyrus was seen. Since the MRI showed drastic improvement, the clinicians opted to perform a lumbar puncture rather than attempting another craniotomy for biopsy. CSF studies showed no gross abnormalities; CSF cytology revealed only lymphocytes (Table 2). Flow cytometry was attempted, but not enough hematopoietic cells were collected for analysis.

**Table 2:** Cerebrospinal fluid laboratory results with reference ranges included Abnormal results are in bold.

Lab	Reference range	Patient value
Color	-	Colorless
Appearance	-	Clear
Nucleated cells	0-5 cells/µL	4
Red blood cells	0 cells/µL	1
Xanthochromia	Absent	Absent
Glucose	40-70 mg/dL	51
Protein	15-45 mg/dL	30

**Figure 7:**
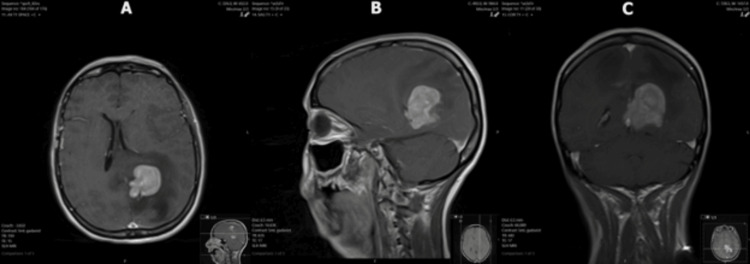
T1 post-contrast MRI images demonstrating significant interval enlargement of the left cingulate gyrus/frontoparietal lesion with surrounding vasogenic edema and new left-to-right midline shift (A) Axial, (B) sagittal, and (C) coronal views of the left cingulate gyrus/frontoparietal lesion, which is enlarged from prior imaging, with new vasogenic edema, mass effect, and mild left transtentorial herniation. MRI: magnetic resonance imaging

**Figure 8:**
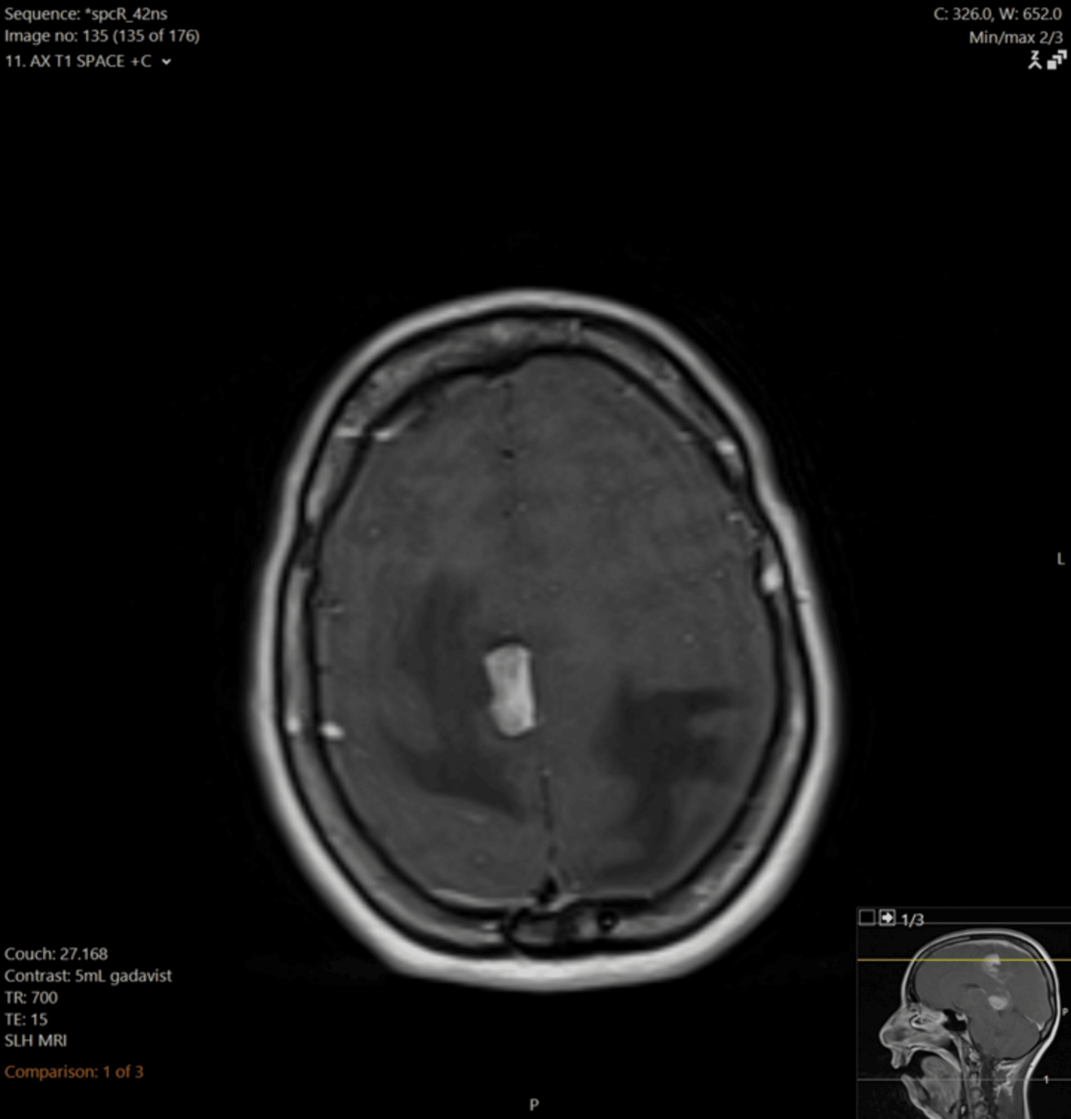
T1 post-contrast MRI image with interval enlargement of the right frontoparietal lesion with adjacent vasogenic edema MRI: magnetic resonance imaging

The response of the lesions to steroids led the radiologists and primary team to favor a diagnosis of PCNSL, but a definitive diagnosis could not be made without pathology. Dexamethasone was discontinued over concerns that steroids were interfering with the results of the previous biopsy and CSF studies. Another MRI and craniotomy were scheduled to be performed two months later. When the patient presented for the second biopsy, she complained of worsening confusion and ataxia. This third MRI - performed 120 days after the original MRI - demonstrated significant interval enlargement of the enhancing lesion in the left cingulate gyrus, now extending into the left frontoparietal region (Figure 7). It also showed recurrence of the right frontoparietal lesion with significant surrounding vasogenic edema (Figure 8). Additionally, a new left-to-right midline shift and left-sided transtentorial herniation were noted on this exam. Based on these results, a second biopsy and lumbar puncture were performed. Repeat CSF studies demonstrated elevated protein (Table 3). Flow cytometry of the biopsied lesion found atypical CD10-positive B-cells. Biopsy confirmed a final diagnosis of diffuse large B-cell lymphoma. The sampled tissue was CD20, BCL-2, and BCL-6-positive.

**Table 3:** Repeated cerebrospinal fluid laboratory results after discontinuing steroids with reference ranges included Abnormal results are in bold. EBV: Epstein-Barr virus; PCR: polymerase chain reaction

Lab	Reference range	Patient value
Color	-	Colorless
Appearance	-	Clear
Nucleated cells	0-5 cells/µL	4
Red blood cells	0 cells/µL	0
Xanthochromia	Absent	Absent
Glucose	40-70 mg/dL	50
Protein	15-45 mg/dL	117
EBV PCR	Not detected	Not detected

## Discussion

This case illustrates the difficulties associated with obtaining a definitive diagnosis of PCNSL in clinical practice. The differential diagnosis for a patient with a subacute onset of neurologic deficits includes infectious, metabolic, demyelinating, autoimmune, vascular, and neoplastic processes. A prompt initiation of treatments can halt disease progression, alleviate symptoms, and protect against chronic neurologic disabilities. The threshold to start these medications is generally low, especially when CT imaging fails to provide a clear and obvious diagnosis in the emergency department. In this case, dexamethasone and levetiracetam were started prophylactically to potentially treat a demyelinating process or seizure. This patient’s initial neuroimaging showed multiple masses, vasogenic edema, and midline shift. Therefore, dexamethasone treatment was also indicated to reduce neuroinflammation and intracranial pressure. However, steroids are also known to induce tumor lysis and regression of lymphoma. Even short-term use has been associated with false-negative or inconclusive biopsy results [23-25]. Physicians often face a difficult decision when considering steroids in patients with brain masses and edema; treatment is often necessary to decrease intracranial swelling, but it also can obscure and delay a final pathology diagnosis in cases of PCNSL.

The findings of the initial MRI favor a diagnosis of PCNSL. The presence of multiple masses in both cerebral hemispheres, with further extension into the deep brain structures with subependymal spread, is classically seen in CNS lymphoma. Involvement of the corpus callosum is common in PCNSL and narrows the differential diagnosis. While PCNSL more commonly presents as a single mass, multiple lesions are not uncommon and have been reported in 34% of cases [26,27]. The homogenous post-contrast enhancement pattern, restricted diffusion, and mass effect reported on MRI helped to rule out a demyelinating process like multiple sclerosis. The locations of these masses, homogenous contrast enhancement, sharp borders, subependymal involvement, and a lack of necrosis all favor a diagnosis of PCNSL over glioma, but other CNS neoplasms could not be excluded with MRI alone [28]. MRS was performed to better understand the metabolic composition of these lesions.

The presence of a normal myoinositol peak compared to the contralateral normal favors a diagnosis of CNS lymphoma compared to glioma [20]. The drastic elevation of the choline-to-creatinine ratio and mildly decreased N-acetylaspartate of the lesion is also more suggestive of PCNSL, rather than glioma or demyelination [18,19]. The large elevation in the lipid peak observed at 30 ms is also significantly linked with PCNSL [17,20]. In this case, the MRS interpretation clearly favored a diagnosis of PCNSL compared to glioma or a tumefactive demyelinating process.

Continued steroid treatment was indicated in this case to reduce the burden of cerebral edema. However, the continuation of steroids likely contributed to a non-diagnostic biopsy. The second MRI demonstrated almost complete regression of the previously described lesions, delaying a second biopsy. CSF studies were also within normal limits. After steroids were discontinued, the patient reported worsening symptoms. The CNS lesions rapidly enlarged as evidenced by the third MRI, with greater vasogenic edema and mass effect compared to the first MRI. Biopsy and flow cytometry provided a final diagnosis of diffuse large B-cell lymphoma, but diagnosis and targeted therapy were unfortunately delayed by several months due to continued suppression of the lesions with steroids. Histopathology after stereotactic brain biopsy remains the gold standard for diagnosing PCNSL; the sensitivity of CSF cytology remains poor but can be improved significantly with flow cytometry [6-8]. While guidelines warn against steroid administration to prevent false-negative biopsies, this recommendation is difficult to effectuate in clinical practice because steroids may be indicated to decrease inflammation.

This case highlights the efficacy of MRS in diagnosing CNS neoplasms. MRS can be performed more promptly and is non-invasive compared to stereotactic biopsy. The utilization of MRS can help alleviate the dilemma that physicians may face when a future biopsy is needed to diagnose PCNSL, but steroids are also integral to protecting the CNS from elevated intracranial pressures. In theory, MRI and MRS can be performed and analyzed shortly after presentation, which can influence the risk-benefit analysis that clinicians use when deciding whether a patient requires a biopsy or steroids. Physicians caring for patients with features of PCNSL on both MRI and MRS may decide on early biopsy without prolonged steroids, even in cases with cerebral edema. Larger studies are required to establish the sensitivity and specificity of MRS in diagnosing different brain tumors. Greater standardization and consensus in MRS will make this tool more reliable in diagnosing cancer on an individual basis. If large studies can replicate consistent and reliable quantitative differences in the metabolite compositions of various CNS neoplasms, it may be possible to approach the diagnosis of PCNSL with a combination of MRI and MRS in the future. A prompt imaging diagnosis would reduce the risk of further dissemination and decrease delays in initiating targeted chemotherapy, thus improving patient outcomes. Even if improved sensitivity and specificity of MRS are achievable, biopsy is still necessary to provide a definitive pathology diagnosis and crucial immunohistochemistry insights. While the results of this single case cannot be generalized to make bold claims, this case report opens a discussion on the diagnostic possibilities of MRS. Greater utilization and study of this imaging modality will help radiologists more confidently diagnose CNS pathologies including PCNSL and could possibly alter the diagnosis and early management of brain lesions in the future.

## Conclusions

PCNSL is a rare but aggressive neoplasm. Diagnosis of CNS lesions can prove difficult; PCNSL typically requires a stereotactic brain biopsy for a definitive diagnosis. Steroids are often administered prophylactically to patients with new neurologic symptoms and to reduce cerebral edema when mass effect is appreciated on neuroimaging. However, steroids are known to cause regression of PCNSL lesions, contributing to false-negative biopsies and delaying the initiation of targeted chemotherapy. This case highlights the classic MRI findings of PCNSL lesions. MRS analysis demonstrated a metabolic profile consistent with PCNSL. A histopathological diagnosis was delayed by several months, as continued steroid administration likely contributed to a false-negative biopsy, cytology, and flow cytometry. Avoiding delays in diagnosing aggressive neoplasms like PCNSL is crucial so patients can begin treatment sooner. More research in the field of MRS is needed to validate its efficacy in differentiating various CNS lesions. If greater standardization in MRS is achieved, an imaging diagnosis of PCNSL with MRI and MRS correlation may be possible in the future in situations where stereotactic biopsy is considered invasive or unfeasible, or in the case of a non-diagnostic biopsy. This case cautions the use of long-term steroids in cases where PCNSL is suspected. MRI and MRS results indicative of PCNSL may correctly influence physicians to expedite biopsy and avoid steroids for patients with cerebral edema.
